# High time to omit oxygen therapy in ST elevation myocardial infarction

**DOI:** 10.1186/s12873-018-0187-0

**Published:** 2018-10-20

**Authors:** Ardavan Khoshnood

**Affiliations:** 1 Department of Clinical Sciences, Faculty of Medicine, Lund University, Lund, Sweden; 2 Department of Emergency and Internal Medicine, Skåne University Hospital Lund, Lund, Sweden

**Keywords:** Oxygen, Oxygen therapy, ST elevation myocardial infarction, STEMI, Physiology, Pathology, Emergency medicine

## Abstract

Supplemental oxygen (O_2_) therapy in patients with chest pain has been a cornerstone in the treatment of suspected myocardial infarction (MI). Recent randomized controlled trials have, however, shown that supplemental O_2_ therapy has no positive nor negative effects on cardiovascular functions, mortality, morbidity or pain in normoxic patients with suspected MI and foremost patients with ST Elevation Myocardial Infarction (STEMI). O_2_ therapy in normoxic STEMI patients should therefore be omitted. More studies are needed in discussing hemodynamically unstable STEMI patients, as well as patients with non-STEMI, unstable angina and other emergency conditions.

## Main text

ST Elevation Myocardial Infarction (STEMI) is the most serious manifestation of Acute Coronary Syndrome (ACS) and is by the Fourth Universal Definition of Myocardial Infarction deemed to be a Type I myocardial infarction, caused by an acute atherothrombotic coronary artery disease (CAD) [1]. The consequence of CAD is a partial or complete occlusion of a coronary artery thus contributing to the termination of oxygen (O_2_) supply to the myocardium, giving rise to ischemia [1, 2].

Because of its seriousness, prompt and rapid diagnosis and treatment is of high importance to reduce both mortality and morbidity [3]. The most important treatment in STEMI is Percutaneous Coronary Intervention (PCI), which should be performed as soon as possible after that the condition is diagnosed [4–6].

International guidelines also emphasize on treatment with dual antiplatelets in the emergency phase before the start of the PCI [4–6]. Previous guidelines also recommended the immediate administration of O_2_ to patients with diagnosed or suspected ACS, without any respect to the blood O_2_ saturation [7–10]. In discussing current guidelines, only the 2017 ESC Guidelines for the management of patients with STEMI [5] states that O_2_ should not routinely be administrated to patients with STEMI, and that only those with a blood O_2_ saturation < 90% or PaO_2_ < 60 mmHg should receive O_2_ therapy.

With an overwhelming scientific evidence that O_2_ therapy has no positive (nor negative) effects in patients with STEMI, all international, regional and local guidelines should be updated and omit O_2_ therapy in normoxic (≥ 90%) STEMI patients.

## Oxygen therapy

The history of O_2_ as a medicine dates back to 1775 when the British chemist Joseph Priestly discovered O_2_ and stated that it could be used as a medicine [11]. It was, however, in 1900 that the first publication on the role of O_2_ therapy in patients with chest pain was published. It was a short letter by Dr. Charles Steele, in which he deemed that O_2_ therapy had relieved chest pains in one single patient he believed to have angina [12]. Ever since this letter by Dr. Steele, several studies have tried to answer how supplemental O_2_ therapy in both healthy and ill patients affect their cardiovascular system.

The rationale behind O_2_ therapy has been that by adding O_2_ to the patient’s blood, the myocardium can be oxygenated, which in turn will contribute to a diminished ischemic area and infarct size, thereby minimizing the risk for lethal arrhythmias [13, 14]. Studies on canines [14–16] have given some support to this theory, showing that O_2_ therapy decreases infarct size and ischemia in these animals. A recent study on swine, however, showed that hyperoxemia can aggravate and worsen myocardial ischemia [17].

In healthy individuals, experimental studies have shown that hyperoxemia because of supplemental O_2_ therapy, may contribute to negative cardiovascular effects like a decrease in coronary blood flow, arterial vasoconstriction, diminished cardiac output, an increase in the systemic vascular resistance as well as impaired blood flow to organs and tissues [18–26].

In patients with suspected as well as confirmed myocardial infarction, the role of O_2_ were for a long time highly inconclusive. Our knowledge gap in this matter was not because of lack of studies, but rather because of the poor methodologies used in these studies. Ever since 1900, several studies have been published on the role of O_2_ in patients with chest pain, coronary artery disease, cardiac failure as well as suspected and confirmed myocardial infarction. All of them have unfortunately had serious limitations and have therefore not been able to correctly answer the question of how O_2_ therapy affects the cardiovascular system in both healthy patients and patients with myocardial infarction and cardiac failure. The studies have either been case studies or small reports including only a few patients, thus not being generalizable, or small studies [18–20, 27–43]. Furthermore, the vast majority of the studies was conducted in the pre-PCI era and even before Troponin was used as an important part in the diagnosis of myocardial infarction.

Because of the above limitations, a Cochrane report from 2013 [44] called for randomized controlled trials to once for all answer the question about which role supplemental O_2_ therapy should have in patients with chest pain and suspected myocardial infarction. The authors of the report stated that “A definitive randomised controlled trial is urgently required […].”

## Randomized controlled trials

Before the Cochrane reports call for a definitive randomized controlled trial (RCT) in 2013, there were already four RCTs on the role of supplemental O_2_ in patients with myocardial infarction; Rawles et al. from 1976 [45], Wilson et al. from 1997 [42], Ukholkina et al. from 2005 [46] and Ranchord et al. from 2012 [47].

The two first studies were conducted in the pre-PCI era. While Wilson et al. [42] found no significant differences between the patients with myocardial infarction randomized to supplemental O_2_ or air, Rawles et al. [45] showed that patients with myocardial infarction receiving supplemental O_2_, had a larger infarct size as measured with serum aspartate aminotransferase.

The study by Ukholkina et al. [46] is the only randomized study showing a positive effect of supplemental O_2_ therapy in patients with myocardial infarction. The study is thus highly biased because of a limited methodology [44].

Rancord et al. [47] have a sound methodology, and should be considered the first modern RCT on the role of supplemental O_2_ in patients with STEMI. The authors found no significant differences between the two arms (supplemental O_2_ vs titrated O_2_) with regard to infarct size as measured by cardiac Troponin T, as well as cardiac MRI (CMRI) close to one month after inclusion.

After the Cochrane report from, three more RCTs have been published discussing the role of supplemental O_2_ therapy in myocardial infarction; the AVOID study [48, 49], the SOCCER study [50–53] and the DETO2X study [54–57].

The main publication of the AVOID study was conducted by Stub et al. [48] in which 441 STEMI patients were randomized to supplemental O_2_ therapy or air. Even though the study found no significant difference in infarct size as measured by cardiac Troponin, a subset of the patients undergoing CMRI after six months, showed that those randomized to supplemental O_2_ therapy, had a larger infarct size as measured in absolute mass but not in percent of the left ventricle. A sub study [49] of the AVOID trial showed later that patients randomized to the O_2_ arm, had significantly higher cardiac Troponin rates than those randomized to the air arm.

The SOCCER study was conducted in Sweden by Khoshnood et al. and aimed to evaluate the effects of supplemental O_2_ in normoxic first-time STEMI patients accepted for PCI. Patients were randomized to either supplemental O_2_ therapy or air. All patients underwent CMRI, while only a subset of patients underwent echocardiography. Their chest pain was scored and assessed prehospital and in-hospital with the Visual Analog Scale (VAS) [50]. Ninety-four patients underwent CMRI which showed no significant difference between the two arms in discussing infarct size, myocardium at risk and myocardial salvage index [51]. Of the 87 patients undergoing echocardiography, no significant differences could be measured between the two arms in discussing left ventricular ejection fraction and wall motion score index [52]. In a recently published sub study, 111 patients were assessed in regard to chest pain to evaluate the analgesic effect of O_2_ therapy. Those randomized to the supplemental O_2_ group had significantly higher median VAS and also received significantly higher amounts of morphine. The study could not show that supplemental O_2_ diminished chest pain [53].

The DETO2X study was also conducted in Sweden. The main publication by Hofmann et al. included more than 6000 patients and evaluated the one-year-all-cause mortality in normoxic patients with suspected myocardial infarction randomized to supplemental O_2_ therapy or ambient air. The study found no significant differences between the two arms in regard to mortality nor morbidity [55]. A sub study on patients with only STEMI (*n* = 2807) did not show any significant differences between the two arms in regard to one-year all-cause mortality, or morbidity like myocardial infarction and cardiogenic shock [56]. In a recent published DETO2X sub study by Sparv et al. [57] on the analgesic effect of supplemental O_2_ therapy in patients with suspected myocardial infarction, there were no significant differences between the two arms in regard to pain nor the amount of morphine and sedatives received during PCI.

Table 1 summarizes all the RCTs.

**Table 1:** A summary of the randomized controlled trials studying the effects of O_2_ therapy in patients with suspected or confirmed myocardial infarctions

Author (Year)	Study Design	Outcome	Limitations
Rawles et al. [45] (1976)	Double blind. Inclusion: Suspected MI. Patients randomized to O_2_ or air.	IS increased in patients treated with O_2_ as measured by AST. No significant differences were shown between the arms in discussing mortality, malignant arrythmias and use of analgesics.	Only those with suspected MI was included,why it is uncertain how many who in fact did had a MI. The study was conducted pre-PCI era. IS was measured by AST. No description of how the randomization sequence was conducted.
Wilson et al. [42] (1997)	Open label. Inclusion: Confirmed MI. Patients randomized to O_2_ or air.	No significant differences were shown between the arms in discussing arrhythmias as well as ST segment changes in the ECG.	The study was conducted pre-PCI era. IS was measured by AST. 16% of those initially included, fell out and was thus not analyzed in the final analysis cohort.
Ukholkina et al. [46] (2005)	Open label. Inclusion: Confirmed MI. Patients randomized to O_2_ or air.	MaR, IS and arrhythmias were significantly lower in the O_2_ group.	The randomization process is unclear. Many have been excluded without any discussion. IS was measured by CKMB and through ECG mapping.
Ranchord et al. [47] (2012)	Open label. Inclusion: STEMI/LBBB. Patients randomized to O_2_ or titrated O_2_.	No significant differences between the two arms in discussing IS as measured with cTn and MRI, as well as 30-day mortality.	Data is lacking for a considerable amount of the patients in regard to mortality. MRI was performed in a subgroup of patients surviving more than 30 days, thus giving rise to a possible selection bias.
Stub et al. [48] (2015)	Open label. Inclusion: STEMI. Patients randomized to O_2_ or air.	Patients in the O_2_ group had a significantly higher mean peak CK but not cTn, increased IS as measured with MRI, and a higher rate of arrythmias as well as recurrent MI.	CK is not specific for MI. MRI was conducted in only some patients, thus giving rise to a possible selection bias. MRI showed increased IS measured in grams of the LV, but not as a percentage of the LV.
Nehme et al. [49] (2016)	Sub study. The main study was conducted by Stub et al. (2015).	For every 100 L of O_2_ given to a patient, both cTnI as well as CK, increased with 1.4% and 1.2% respectively.	See limitations for Sub et al. (2015). A little over 8% of the patients were excluded since they had no cTnI measurements.
Khoshnood et al. [51] (2017)	Single blind. Inclusion: STEMI. Patients randomized to O_2_ or air.	No significant differences between the two groups in discussing MSI, MaR and IS.	MRI was conducted in only some patients, thus giving rise to a possible selection bias.
Khoshnood et al. [52] (2017)	Sub study. The main study was conducted by Khoshnood et al. (2017; ref. 44).	No significant differences between the groups in discussing WMSI, LVEF as well as NT-proBNP.	A considerable number of patients were excluded because they, among others, denied participation after that they were initially included. This may be a source of bias.
Khoshnood et al. [53] (2018)	Sub study. The main study was conducted by Khoshnood et al. (2017; ref. 44).	Before the randomization, patients in the O_2_ group had a significantly higher VAS and also received significantly more morphine. No significant differences between the two groups in regard to VAS at the start of the PCI or median VAS decrease from randomization to PCI.	A considerable amount of the patients missed VAS rates and were therefore excluded. This may be a source of bias.
Hoffman et al. [55] (2017)	Open label. Inclusion: Suspected MI. Patients randomized to O_2_ or air.	No significant differences between the groups on all-cause mortality at 1 year.	The study may have been underpowered.
Hoffman et al. [56] (2018)	Sub study. The main study was conducted by Hoffman et al. (2017).	No significant differences between the groups in discussing all-cause mortality at 1 year, or adverse cardiac events like MI rehospitalization or cardiogenic chock.	See limitations for Hoffman et al. (2017).
Sparv et al. [57] (2018)	Sub study. The main study was conducted by Hoffman et al. (2017).	No significant differences between the groups in discussing analgesic effect, or the use of both sedatives and opiates during PCI.	Some of the included patients received opiates in the ambulance, why it may have decreased pain at the PCI.

*AMI* Acute Myocardial Infarction, *AST* Aspartate Transaminase, *CK* Creatine Kinase, *CKMB* Creatine kinase-MB, *cTn* Cardiac Troponin, *cTnI* Cardiac Troponin I, *ECG* Electrocardiogram, *IS* Infarct Size, *LBBB* Left Bundle Branch Block, *LV* Left Ventricle, *LVEF* Left Ventricular Ejection Fraction, *MaR* Myocardium at Risk, *MI* Myocardial Infarction, *MRI* Magnetic Resonance Imaging, *MSI* Myocardial Salvage Index, *O*_*2*_ Oxygen, *PCI* Percutaneous Coronary Intervention, *STEMI* ST Elevation Myocardial Infarction, *VAS* Visual Analog Scale, *WMSI* Wall Motion Score Index

## Omit supplemental O_2_ therapy in STEMI

The above RCTs clearly show that O_2_ therapy has so positive nor negative cardiovascular effects, when used in normoxic patients with STEMI both prehospital and in-hospital. Two recent reviews and meta-analysis on the role of supplemental O_2_ therapy in acute myocardial infarction, showed also no benefit of using O_2_ therapy in these patients [58, 59].

**Fig. 1:**
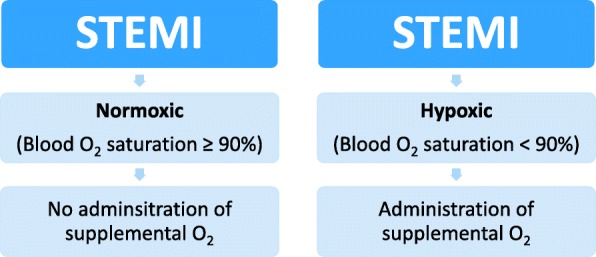
Suggestion on how to manage patients with ST Elevation Myocardial Infarction (STEMI) both in a prehospital setting and in-hospital

In discussing supplemental O_2_ therapy in normoxic STEMI patients, the evidences are clear and consistent, why all guidelines must be reformed to state that supplemental O_2_ therapy in these patients should be omitted. It is, however, of high importance to point that patients diagnosed with STEMI, and who have a low blood oxygen saturation, should receive supplemental O_2_. It is the routine use of O_2_ therapy, with no respect to blood oxygen saturation, that should be omitted (Fig. 1). With this said, it is important to point out that the RCTs presented above does also have some limitations as the majority of them have had a small cohort, and the focus have been stable and normoxic STEMI patients. These limitations might reduce the generalizability of the studies. More studies are therefore needed in discussing supplemental O_2_ therapy in hemodynamic unstable STEMI patients, patients with non-STEMI as well as unstable angina. This is especiCally of importance since some studies argue that supplemental O_2_ therapy administrated to acutely ill patients can be toxic and increase mortality and morbidity [60, 61].

## Abbreviations

ACS: Acute Coronary Syndrome
CAD: Coronary Artery Disease
CMRI: Cardiac Magnetic Resonance Imaging
MI: Myocardial Infarction
O_2_: Oxygen
PCI: Percutaneous Coronary Intervention
RCT: Randomized Controlled Trials
STEMI: ST Elevation Myocardial Infarction
VAS: Visual Analog Scale
